# Assessing the Impact of Optimal Health Education Programs on the Control of Zoonotic Diseases

**DOI:** 10.1155/2020/6584323

**Published:** 2020-07-11

**Authors:** A. Mhlanga

**Affiliations:** Department of Mathematics, University of Zimbabwe, P.O. Box MP 167 Mount Pleasant, Harare, Zimbabwe

## Abstract

To better understand the dynamics of zoonotic diseases, we propose a deterministic mathematical model to study the dynamics of zoonotic brucellosis with a focus on developing countries. The model contains all the relevant biological details, including indirect transmission by the environment. We analyze the essential dynamic behavior of the model and perform an optimal control study to design effective prevention and intervention strategies. The sensitivity analysis of the model parameters is performed. The aim of the controls is tied to reducing the number of infected humans, through health promotional programs within the affected communities. The Pontryagin's Maximum Principle is used to characterize the optimal level of the controls, and the resulting optimality system is solved numerically. Overall, the study demonstrates that through health promotional programs on zoonotic diseases among villagers, it is vital that they should be conducted with high efficacy.

## 1. Introduction

Zoonoses (also known as zoonosis and zoonotic diseases) are infectious diseases caused by bacteria, viruses, and parasites that spread between animals and humans. Some types of zoonotic diseases are African sleeping sickness, anthrax, bird flu, brucellosis, influenza, rabies, Zika, and Ebola [1]. The zoonotic diseases have been categorized into the more common endemic zoonoses such as salmonellosis, brucellosis, and leptospirosis which are responsible for more than 2.2 million human deaths and 2.4 billion cases of illness annually and the less common epidemic and emerging zoonoses such as rift valley, anthrax, valley fever, Ebola, and Zika which either occur in sporadic outbreaks in neglected populations or that are new or reappearing with an increased incidence of geographical range [2]. Zoonotic diseases have several modes of transmission, with the main ones being direct, indirect, and vector-borne transmissions [3]. Direct transmission entails coming into contact with the saliva, blood, urine, mucous, feces, or other bodily fluids. Indirect contact is due to coming into contact with areas where animals live and roam or surfaces that have been contaminated, such as pet habitants, pet food, chicken coops, and aquarium tank water. A vector-borne entails being bitten by a tick or an insect like a mosquito or flea. This class of diseases has been the principal source of emerging health risks, and it is estimated that zoonotic pathogens have accounted for more than 60% of emerging infectious diseases recently [2]. Some of the risk factors associated with the emergence of zoonotic diseases and spill over into humans include human encroachment, population expansion, consumption of exotic food, migratory movements, and ecotourism [4]. Endemic zoonotic diseases have the dual impact of causing illness and death in humans and animals as well as substantial loss in resource-poor societies where livestock farming is a major engine of economic growth at the household and national levels. The World Bank estimated that 6 major zoonotic disease epidemics during 1997-2009 resulted in an economic loss of more than $80 billion [1]. Controlling zoonotic disease outbreaks has become ever more important, it has been estimated that since 1940, about 40% of the emerging infectious diseases affecting humans globally, but mainly in developing countries, have originated from animals, both domestic and wild [5, 6].

The attention given to zoonotic diseases has however focused more on emerging zoonoses that pose global economic and health threats and less on the endemic zoonotic disease which tend to occur among populations with little financial muscle, such as in sub-Saharan Africa. The relative risk for emerging infectious disease vents from wildlife sources has continued to be a challenge in sub-Saharan Africa. An increase in the interaction between wildlife, domestic animals, and humans increases the chance of zoonotic disease transmission. The sub-Saharan region was identified as one of the hotspot regions with a high prevalence of endemic zoonotic diseases and where it has a large rural population that lives in close proximity with livestock and wildlife [6, 7]. In some parts of the sub-Saharan countries such as Zimbabwe, they still have problems regarding the control of zoonoses, mainly due to the lack of enough infrastructure and resources for disease surveillance [7]. Poverty and lack of education towards the zoonotic diseases lead many people, especially those from rural areas, accessing commodities such as fresh unpasteurised milk and uninspected meat from domestic animals on the informal food markets [7]. Some researchers have managed to show that the risk of zoonotic diseases would increase or decrease, in the various keeping systems and to the public as a whole depending on their education levels towards zoonotic diseases. Low levels of education towards zoonotic disease among the rural villagers have been one of the major setbacks in the fight against zoonotic diseases [8]. Historically, infectious disease specialists in collaboration with governmental organizations have attempted to develop effective controls and eradication strategies gradually, using field experience that is unique to the region and disease. A particular challenge in controlling zoonotic infections in this way is to appropriately allocate resources in the multispecies system [8]. Thus, designing effective control strategies requires achieving a proper tradeoff between the costs resulting from disease prevalence and the costs of control.

Mathematical modeling, analysis, and simulation for infectious diseases have proved to be an essential guiding tool that could give a sound direction to policymakers and public health administration on how to effectively prevent and control zoonotic diseases. Mathematical modeling of zoonotic diseases has been a particular area of burgeoning interest over the last few years, see the articles [4, 8–16] for a few representative samples. The current study complements many of the earlier published studies by providing a rigorous qualitative analysis of a mathematical model which seeks to understand the impact of educational campaigns in curtailing the spread of zoonotic diseases. In this paper, we propose a compartmental model for the spread of zoonoses incorporating all the essential biological details. The model incorporates the aspect of educational campaigns in the human-domestic animal interface space and allows optimal control methods to be used. The model also includes direct and indirect modes of transmission. Our study focuses on zoonotic diseases, humans, and domestic animals; hence, we shall make use of brucellosis as a zoonotic disease and cattle as domestic animals, for illustrative purposes. Brucellosis is a zoonotic disease that affects domesticated animals, wildlife, and humans. Animals acquire the infection mainly through direct contact with infected cattle or indirectly from the environment containing large quantities of bacteria discharged by infected individuals [17], whereas in human, common routes of infection include direct inoculation through cuts and abrasions in the skin or inhalation of infectious aerosols and ingestion of infectious unpasteurized milk or other dairy products [18]. Human to human transmission is extremely rare [17, 18].

The structure of the paper is as follows.  Section 2 constitutes model formulation, and analytic results are presented in Section 3. The sensitivity analysis of the reproduction number is reported in Section 4, and the optimal control analysis is presented in Section 5. Numerical simulations are presented in Section 6. The paper concludes with a discussion in Section 7.

## 2. Model Formulation

In this section, we introduce a continuous mathematical model for the transmission dynamics of zoonotic disease in the form of brucellosis in both humans and cattle. Guided by the information on the natural history of brucellosis infection in both humans and cattle populations to determine, the basic plausible assumptions for the model formulation are determined [19, 20]. The human population is divided into four mutually exclusive epidemiological subpopulations consisting of uneducated susceptibles (*S*_*h*_), educated susceptibles (*C*), infected (*I*_*h*_), and the recovered (*R*_*h*_) humans, so that the total human population is given by (*N*_*h*_), where *N*_*h*_ = *S*_*h*_ + *C* + *I*_*h*_ + *R*_*h*_. The cattle population is divided into three mutually exclusive epidemiological subpopulations consisting of the susceptible (*S*_*a*_), infected (*I*_*a*_), and recovered (*R*_*a*_) cattle, so that the total cattle population is given by *N*_*a*_ = *S*_*a*_ + *I*_*a*_ + *R*_*a*_. We denote the quantity of the brucella in the environment by *W*, which is shed off at a rate *δ*_*a*_ by the cattle and *δ*_*h*_ by humans. We assume that the brucella in the environment decays at the rate of *r*. The natural death rates for the humans and cattle are given by *μ*_*h*_ and *μ*_*a*_, respectively. The recruitment of the susceptible humans and the susceptible cattle are through birth, given by *Λ*_*h*_ and *Λ*_*a*_, respectively. We assume homogeneous mixing, that is, all the susceptible humans have the same likelihood to be infected and also the susceptible cattle have the same chance to be infected. The force of infection for humans is given by

(1) $$\begin{array}{c} \lambda_{h}=\frac{\beta_{h}I_{a}}{N_{a}}+\frac{\beta_{w_{h}}W}{K}, \end{array}$$

where *β*_*h*_ is the effective contact rate by the infected cattle towards humans. *β*_*w*_*h*__ is the indirect infection from the brucella in the environment to the susceptible humans. *K* denotes the brucella concentrations measured with respect to their infection doses. The force of infection for the cattle is given by

(2) $$\begin{array}{c} \lambda_{a}=\frac{\beta_{a}I_{a}}{N_{a}}+\frac{\beta_{w_{a}}W}{K}, \end{array}$$

where *β*_*a*_ is the effective contact rate by the infected cattle on the susceptible cattle. *β*_*w*_*a*__ is the indirect infection from the brucella in the environment to the susceptible cattle. The uneducated susceptible individuals are educated towards zoonotic diseases at a rate of *θ* through educational campaigns in the form of health promotional programs. A parameter *η* represents the efficacy of health promotion programs. If *η* = 0, then the health promotion programs are not effective, if *η* = 1 corresponds to completely effective health promotion programs, while 0 < *η* < 1 implies that the health promotion programs will be effective to some degree. Thus, the susceptible uneducated humans are infected with brucellosis at a rate of *λ*_*h*_ while the susceptible educated humans are infected at a rate of (1 − *η*)*λ*_*h*_. We assume that the infected humans recover from the brucellosis at a rate of *γ* while the cattle recover at a rate of *ϕ*, both getting permanent immunity [18, 21]. Parameter *v* represents death due to the respective zoonotic disease, and *τ* represents the culling rate. We assume that the rate of culling is at its minimal since most of the villagers in sub-Saharan Africa on the human-domestic animal-wildlife interface are not well educated in terms of dealing with zoonotic diseases. We shall refer to the health promotional programs as educational campaigns, throughout the manuscript. Here, we construct a system of nonlinear differential equations to model the disease dynamics of brucellosis.

(3) $$\begin{array}{c} \left\{\begin{array}{c} S_{h}^{\prime }=\Lambda_{h}-\lambda_{h}S_{h}-\theta S_{h}-\mu_{h}S_{h},\\ C^{\prime }=\theta S_{h}-\left(1-\eta \right)\lambda_{h}C-\mu_{h}C,\\ I_{h}^{\prime }=\lambda_{h}S_{h}+\left(1-\eta \right)\lambda_{h}C-\left(\mu_{h}+\gamma \right)I_{h},\\ R_{h}^{\prime }=\gamma I_{h}-\mu_{h}R_{h},\\ S_{a}^{\prime }=\Lambda_{a}-\lambda_{a}S_{a}-\mu_{a}S_{a},\\ I_{a}^{\prime }=\lambda_{a}S_{a}-\left(\mu_{a}+\phi +v+\tau \right)I_{a},\\ R_{a}^{\prime }=\phi I_{a}-\mu_{a}R_{a},\\ W^{\prime }=\delta_{h}I_{h}+\delta_{a}I_{a}-rW. \end{array}\right. \end{array}$$

The model variables and their descriptions are summarised in Table 1.

The model parameters and their possible values are shown in Table 2.

## 3. Analysis of the Model

### 3.1. Positivity and Boundedness of Solutions

It can be easily proved that the domain of biological interest

(4) $$\begin{array}{c} G=\left\{S_{h}\left(t\right),C\left(t\right),I_{h}\left(t\right),R_{h}\left(t\right),S_{a}\left(t\right),I_{a}\left(t\right),R_{a}\left(t\right),W\left(t\right)\text{: }N_{h}\leq \frac{\Lambda_{h}}{\mu_{h}},N_{a}\leq \frac{\Lambda_{a}}{\mu_{a}},W\leq \frac{\delta_{h}\Lambda_{h}+\delta_{a}\Lambda_{\text{a}}}{r\;\min\;\left(\mu_{h},\mu_{a}\right)}\right\} \end{array}$$

is positively invariant and attracting with respect to the model in equation (3).

### 3.2. The Disease-Free Equilibrium and Basic Reproduction Number

It can be established that system (3) always has a disease-free equilibrium (DFE) given by

(5) $$\begin{array}{c} E^{0}=\left(S^{0},C^{0},I_{h}^{0},R_{h}^{0},S_{a}^{0},I_{a}^{0},R_{a}^{0},W^{0}\right)=\left(\frac{\Lambda_{h}}{\theta +\mu_{h}},\frac{\theta \Lambda_{h}}{\mu_{h}\left(\theta +\mu_{h}\right)},0,0,\frac{\Lambda_{a}}{\mu_{a}},0,0,0\right). \end{array}$$

Denoting the reproduction number by *ℛ*_0_, which is a measure of the average number of secondary infections generated by a single infectious case in a fully susceptible population during its infectious period [26], the reproduction number is commonly regarded as a threshold quantity for the disease dynamics, essential in determining the transmission and spread of the disease. Using the next-generation matrix notations in [26], the nonnegative matrix *F* that denotes the generation of new infections and the nonsingular matrix *V* that denotes the disease transfer among compartments are, respectively, given by

(6) $$\begin{array}{c} F=\left[\begin{array}{ccc} 0 & \frac{\left(\left(1-\eta \right)\theta +\mu_{h}\right)\mu_{a}\beta_{h}\Lambda_{h}}{\left(\theta +\mu_{h}\right)\mu_{h}\Lambda_{a}} & \frac{\left(\left(1-\eta \right)\theta +\mu_{h}\right)\beta_{w_{h}}\Lambda_{h}}{\left(\theta +\mu_{h}\right)\mu_{h}K}\\ 0 & \beta_{a} & \frac{\beta_{w_{a}}\Lambda_{a}}{\mu_{a}K}\\ 0 & 0 & 0 \end{array}\right],V=\left[\begin{array}{ccc} \mu_{h}+\gamma & 0 & 0\\ 0 & \mu_{a}+\gamma +v+\tau & 0\\ -\delta_{h} & -\delta_{a} & r \end{array}\right]. \end{array}$$

That is, the reproduction number *ℛ*_0_ of system (3) is the spectral radius of the next-generation matrix *FV*^−1^,

(7) $$\begin{array}{c} R_{0}=\rho \left(FV^{-1}\right)=\frac{A_{11}+A_{22}+\sqrt{{\left(A_{11}-A_{22}\right)}^{2}+4A_{12}A_{21}}}{2}, \end{array}$$

where

(8) $$\begin{array}{c} A_{11}=\frac{\left(\left(1-\eta \right)\theta +\mu_{h}\right)\delta_{h}\beta_{w_{h}}\Lambda_{h}}{\mu_{h}rK\left(\gamma +\mu_{h}\right)\left(\theta +\mu_{h}\right)},A_{12}=\frac{\left(\left(1-\eta \right)\theta +\mu_{h}\right)\left(\beta_{w_{h}}\delta_{a}\Lambda_{a}+\beta_{h}\mu_{a}rK\right)\Lambda_{h}}{\mu_{h}rK\left(\theta +\mu_{h}\right)\left(\phi +\mu_{a}+v+\tau \right)\text{s}\Lambda_{a}},A_{21}=\frac{\delta_{h}\beta_{w_{a}}\Lambda_{a}}{\mu_{a}rK\left(\gamma +\mu_{h}\right)},A_{22}=\frac{\mu_{a}\beta_{a}rK+\delta_{a}\beta_{w_{a}}\Lambda_{a}}{\mu_{a}rK\left(\phi +\mu_{a}+v+\tau \right)}. \end{array}$$

Making use of Theorem 2 in Van den Driessche and Watmough [26], we establish the following result.

**Theorem 1 .**

If *ℛ*_0_ < 1, then *ℰ*^0^ is locally asymptotically stable and unstable otherwise.

Furthermore, a stronger result regarding the global dynamics of the DFE can be established. We will utilize the approach of Lyapunov functions [27–31] in the analysis of global asymptotic stability.

**Theorem 2 .**

If *ℛ*_0_ ≤ 1, the DFE is globally asymptotically stable in *𝒢*. If *ℛ*_0_ > 1, the system is uniformly persistent.

**Proof**

Let *ℋ*(*t*) = (*I*_*h*_(*t*), *I*_*a*_(*t*), *W*(*t*))^*T*^. Since from (3)

(9) $$\begin{array}{c} \left\{\begin{array}{c} {I_{h}}^{\prime }=\lambda_{h}S_{h}+\left(1-\eta \right)\lambda_{h}C-\left(\mu_{h}+\gamma \right)I_{h},\\ {I_{a}}^{\prime }=\lambda_{a}S_{a}-\left(\mu_{h}+\phi +v+\tau \right)I_{a},\\ W^{\prime }=\delta_{h}I_{h}+\delta_{a}I_{a}-rW, \end{array}\right. \end{array}$$

it follows that

(10) $$\begin{array}{c} \dot{H}\left(t\right)\leq \left(F-V\right)H, \end{array}$$

where *F* and *V* are as defined in equation (6). It is worth noting that *F* and *V*^−1^ are nonnegative. By the Perron-Frobenius Theorem [32], the nonnegative matrix *V*^−1^*F* has a positive left eigenvector *u* with respect to the eigenvalue *ℛ*_0_ = *ρ*(*V*^−1^*F*) = *ρ*(*FV*^−1^); that is, *u*^*T*^*V*^−1^*F* = *ℛ*_0_*u*^*T*^. Motivated by [30], consider a Lyapunov function

(11) $$\begin{array}{c} Y\left(t\right)=u^{T}V^{-1}H. \end{array}$$

Differentiating *𝒴*(*t*) along with solutions of (3), we have

(12) $$\begin{array}{c} \dot{Y}\left(t\right)=u^{\text{T}}V^{-1}\dot{H}\leq u^{T}V^{-1}\left(F-V\right)H=\left(R_{0}-1\right)u^{T}H\leq 0\text{ if }R_{0}\leq 1. \end{array}$$

If *ℛ*_0_ < 1, the equality $\dot{𝒴}\left(t\right)=0$ implies that *u*^*T*^*ℋ* = 0. This leads to *I*_*h*_ = *I*_*a*_ = *W* = 0 since *u* denotes a positive left Perron eigenvector. Hence, when *ℛ*_0_ < 1, system (3) yields *S*_*h*_ = *S*_*h*_^0^, *C* = *C*^0^, *S*_*a*_ = *S*_*a*_^0^, and *I*_*h*_ = *R*_*h*_ = *I*_*a*_ = *R*_*a*_ = *W* = 0. Thus, the invariant set on which $\dot{𝒴}\left(t\right)=0$ contains only the point *ℰ*^0^. If *ℛ*_0_ = 1, we also have $\dot{𝒴}\left(t\right)=0$, and it can also be shown that the invariant set on which $\dot{𝒴}\left(t\right)$ contains only the point *ℰ*^0^. Therefore, by LaSalle's invariance principle [33], *ℰ*^0^ is globally asymptotically stable in *𝒢* when *ℛ*_0_ ≤ 1.

If *ℛ*_0_ > 1, then by continuity, $\dot{𝒴}\left(t\right)>0$ in a neighbourhood of *ℰ*^0^ in the interior of *𝒢*. Solutions in the interior of *𝒢* sufficiently close to *ℰ*^0^ move away from the DFE implying that the DFE is unstable. This completes the proof.

The result established in Theorem 2 portrays that *ℛ*_0_ = 1 is a sharp threshold for disease dynamics: the disease will die out when *ℛ*_0_ ≤ 1, whereas the disease will persist when *ℛ*_0_ > 1. Biologically, a uniform persistent system shows that the infection persists for a long period of time. Now, we investigate uniform persistence, and we claim the following result.

**Theorem 3 .**

If *ℛ*_0_ > 1, system (3) is uniformly persistent, namely, there exists a constant *ζ* > 0 such that

(13) $$\begin{array}{c} \underset{t\to \infty }{\lim}\inf S_{h}\left(t\right)>\zeta ,\underset{t\to \infty }{\lim}\inf C\left(t\right)>\zeta ,\underset{t\to \infty }{\lim}\inf I_{h}\left(t\right)>\zeta ,\underset{t\to \infty }{\lim}\inf R_{h}\left(t\right)>\zeta ,\underset{t\to \infty }{\lim}\inf S_{a}\left(t\right)>\zeta ,\underset{t\to \infty }{\lim}\inf I_{a}\left(t\right)>\zeta ,\underset{t\to \infty }{\lim}\inf R_{a}\left(t\right)>\zeta ,\underset{t\to \infty }{\lim}\inf W\left(t\right)>\zeta , \end{array}$$

for any initial conditions satisfying

(14) $$\begin{array}{c} S_{h}\left(0\right)\geq 0,C\left(0\right)\geq 0,I_{h}\left(0\right)\geq 0,R_{h}\left(0\right)\geq 0,S_{a}\left(0\right)\geq 0,I_{a}\left(0\right)\geq 0,R_{a}\left(0\right)\geq 0,W\left(0\right)\geq 0. \end{array}$$

**Proof**

Let *X* = *𝒢*, *x* = (*S*_*h*_, *C*, *I*_*h*_, *R*_*h*_, *S*_*a*_, *I*_*a*_, *R*_*a*_, *W*) and *X*_0_ = {*x* ∈ *X* | *I*_*h*_ + *I*_*a*_ + *W* > 0}. Hence, *∂X*_0_ = *X*\*X*_0_ = {*x* ∈ *X* | *I*_*h*_ = *I*_*a*_ = *W* = 0}. Let *ψ*_*t*_ be a semiflow induced by the solutions of system (3) and *M*_*∂*_ = {*x* ∈ *∂X*_0_ | *ψ*_*t*_*x* ∈ *∂X*_0_, *t* ≥ 0}. By Equation (4), we have *ψ*_*t*_*X*_0_ ⊂ *X*_0_ and *ψ*_*t*_ is bounded in *X*_0_. Therefore, there exist a global attractor for *ψ*_*t*_. The disease-free equilibrium is the unique equilibrium on the manifold *∂X*_0_ and is globally asymptotically stable on *∂X*_0_. Moreover, ⋃_*x*∈*M*_*∂*__*ω*(*x*) = {*ℰ*^0^} and no subsets of *M* forms a cycle in *∂X*_0_. Finally, since the disease-free equilibrium is unstable on *X*_0_ if *ℛ*_0_ > 1, we deduce that system (3) is uniformly persistent by using a result from [34] (Theorem 1.3.1 and Remark 1.3.1). This completes the proof.

### 3.3. Endemic Equilibrium

System (3) has the endemic equilibrium point given by

(15) $$\begin{array}{c} E^{*}=\left(\begin{array}{c} S_{h}^{*}=\frac{\Lambda_{h}}{\lambda_{h}^{*}+\mu_{h}+\theta },C=\frac{\theta \Lambda_{h}}{\left(\lambda_{h}^{*}+\mu_{h}+\theta \right)\left[\left(1-\eta \right)\lambda_{h}^{*}+\mu_{h}\right]},I_{h}^{*}=\frac{\lambda_{h}^{*}\Lambda_{h}\left[\left(1-\eta \right)\left(\lambda_{h}^{*}+\theta \right)+\mu_{h}\right]}{\left(\lambda_{h}^{*}+\mu_{h}+\theta \right)\left(\gamma +\mu_{h}\right)\left[\left(1-\eta \right)\lambda_{h}^{*}+\mu_{h}\right]},\\ I_{h}^{*}=\frac{\gamma \lambda_{h}^{*}\Lambda_{h}\left[\left(1-\eta \right)\left(\lambda_{h}^{*}+\theta \right)+\mu_{h}\right]}{\mu_{h}\left(\lambda_{h}^{*}+\mu_{h}+\theta \right)\left(\gamma +\mu_{h}\right)\left[\left(1-\eta \right)\lambda_{h}^{*}+\mu_{h}\right]},S_{a}^{*}=\frac{\Lambda_{a}}{\lambda_{a}^{*}+\mu_{a}},I_{a}^{*}=\frac{\lambda_{a}^{*}\Lambda_{a}}{\left(\mu_{a}+\phi +v+\tau \right)\left(\lambda_{a}^{*}+\mu_{a}\right)},\\ R_{a}^{*}=\frac{\phi \lambda_{a}^{*}\Lambda_{a}}{\mu_{a}\left(\mu_{a}+\phi +v+\tau \right)\left(\lambda_{a}^{*}+\mu_{a}\right)},\\ W^{*}=\frac{\delta_{a}\lambda_{a}^{*}\Lambda_{a}\left(\gamma +\mu_{h}\right)\left(\lambda_{h}^{*}+\mu_{h}+\theta \right)\left[\left(1-\theta \right)\lambda_{h}^{*}+\mu_{h}\right]+\delta_{h}\lambda_{h}^{*}\Lambda_{h}\left(\mu_{a}+\phi +v\right)\left(\lambda_{a}^{*}+\mu_{a}\right)\left[\left(1-\eta \right)\left(\lambda_{h}^{*}+\theta \right)+\mu_{h}\right]}{r\left(\mu_{a}+\phi +v+\tau \right)\left(\lambda_{a}^{*}+\mu_{a}\right)\left(\gamma +\mu_{h}\right)\left(\lambda_{h}^{*}+\mu_{h}+\theta \right)\left[\left(1-\eta \right)\lambda_{h}^{*}+\mu_{h}\right]}, \end{array}\right. \end{array}$$

in terms of the forces of infection *λ*_*h*_^∗^ and *λ*_*a*_^∗^. Next, we present the local stability of *ℰ*^∗^ when the reproduction number *ℛ*_0_ is sufficiently close to 1. We shall make use of the Centre Manifold Theory [35], but firstly, we present the following Lemma.

**Lemma 1 .**

Consider the following general system of ordinary differential equations with a parameter *ϕ*:

(16) $$\begin{array}{c} \frac{dx}{dt}=f\left(x,\phi \right),\text{with }f:{\mathbb{R}}^{n}\times \mathbb{R}⟶\mathbb{R}\text{ and }f\in {\mathbb{C}}^{2}\left({\mathbb{R}}^{n}\times \mathbb{R}\right), \end{array}$$

where 0 is an equilibrium of the system, that is *f*(0, *ϕ*) = 0∀ *ϕ*, and assume

**A1.**

*A* = *D*_*x*_ *f*(0, 0) = ((*∂f*_*i*_/*∂x*_*j*_)(0, 0)) is the linearisation matrix of system (3) around the equilibrium 0 and *ϕ* evaluated at 0. Zero is a simple eigenvalue of *A*, and all other eigenvalues of *A* have negative real parts.

**A2.**

Matrix *A* has a right eigenvector *u* and a left eigenvector *v* corresponding to the zero eigenvalue. Let *f*_*k*_ be the *k*^th^ component of *f* and

(17) $$\begin{array}{c} a=\overset{n}{\underset{k,i,j=1}{\sum }}v_{k}u_{i}u_{j}\frac{\partial^{2}f_{k}}{\partial x_{i}\partial x_{j}}\left(0,0\right),\\ b=\overset{n}{\underset{k,i=1}{\sum }}v_{k}u_{i}\frac{\partial^{2}f_{k}}{\partial x_{i}\partial \phi }\left(0,0\right). \end{array}$$

The local dynamics of (16) around zero is totally governed by *a* and *b*.

- *a* > 0, *b* > 0. When *ϕ* < 0 with ∣*ϕ* | <<1, 0 is locally asymptotically stable, there exists a positive unstable equilibrium. When 0 < *ϕ* < <1, 0 is unstable and there exists a negative and locally asymptotically stable equilibrium
- *a* < 0, *b* < 0. When *ϕ* < 0 with ∣*ϕ* | <<1, 0 is unstable; when 0 < *ϕ* < <1, 0 is locally asymptotically stable, and there exists a positive unstable equilibrium
- *a* > 0, *b* < 0. When *ϕ* < 0 with ∣*ϕ* | <<1, 0 is unstable, and there exists a locally asymptotically stable negative equilibrium; when 0 < *ϕ* < <1, 0 is stable, and a positive unstable equilibrium exists
- *a* < 0, *b* > 0. When *ϕ* changes from negative to positive, 0 changes its stability from stable to unstable. Correspondingly, a negative unstable equilibrium becomes positive and locally asymptotically stable

**Theorem 4 .**

The endemic equilibrium point *ℰ*^∗^ is locally asymptotically stable if *ℛ*_0_ > 1 and sufficiently close to 1.

The proof of Theorem 5 is outlined in the appendix.

## 4. Sensitivity Analysis

In this section, we shall perform some sensitivity analysis on our reproduction number. Sensitivity analysis tells us how important each parameter is to disease transmission. Such information is crucial not only for experimental design but also for data assimilation and reduction of complex nonlinear models [36]. Sensitivity analysis is commonly used to determine the robustness of model predictions to parameter values since there are usually errors in data collection and presumed parameter changes. It is used to discover parameters that have a high impact on *ℛ*_0_ and should be targeted by intervention strategies.

Following Arriola and Hyman [37], we present the normalized forward sensitivity indices of *ℛ*_0_ to our model parameters in Table 2. The sensitivity index for *θ*, for example, is

(18) $$\begin{array}{c} Y_{\theta }^{R_{0}}=\frac{\partial R_{0}}{\partial \theta }\times \frac{\theta }{R_{0}}=-0.792678. \end{array}$$

The detailed sensitivity indices of *ℛ*_0_ resulting from the evaluation to other model parameters are shown in Table 3.

The parameters that result in positive index increase the value of *ℛ*_0_ whenever they are increased while those with a negative index decrease the value of *ℛ*_0_ whenever they are increased. For example, since *Y*_*θ*_^*R*_0_^ = −0.792678, increasing the rate at which individuals are educated on zoonotic diseases by 10% results in the decrease of the reproduction number by 7.9%. Thus, educating individuals on zoonotic diseases would be crucial in curtailing the spread of brucellosis. Similarly, improving the efficacy of the educational campaigns by 10% would also trigger a reduction of the reproduction number by 4%. It is worth noting that the culling of the infected animals would be beneficial, since its sensitivity index is given by *τ* = −0.504128. Thus, culling and burning of carcasses should be carried out, since it had also been noted by some researchers that individuals eat infected animals due to lack of sufficient and correct information, regarding the spread or infectiousness of zoonotic diseases [7].

## 5. Optimal Control

We noted that for *ℛ*_0_ > 1, brucellosis becomes an endemic epidemic. Thus, it is of our interest to explore effective control strategies against this zoonotic disease. In sub-Saharan Africa, individuals who live around the frontiers of human-domestic animal-wildlife interface areas do not have enough awareness and education on zoonotic diseases like brucellosis and bovine TB [7]. Thus, implementation of the health promotion programs in the form of educational campaigns could play an important role in controlling zoonotic diseases among the human population.

We now reconsider model system (3), and we introduce two time-dependent controls in *u*_1_(*t*) and *u*_2_(*t*). The control effort *u*_1_(*t*) models optimal educational campaigns, and the control *u*_2_(*t*) increases the positive impact of the educated individuals from being infected by zoonotic diseases. It is advisable to note that when the educational campaigns are strengthened with higher efficacy, the infection risk will be reduced. At the same time, we hope to minimize the costs of achieving this. Therefore, in this section, we will perform a study on the optimal design of the educational campaigns to control the transmission and the spread of brucellosis, using optimal control theory [38–40]. Educational campaigns, in this case, are aimed at encouraging the uninfected to have some protective behaviors.

Thus, introducing two-time dependent controls *u*_1_(*t*) and *u*_2_(*t*), system (3) now becomes

(19) $$\begin{array}{c} \left\{\begin{array}{c} S_{h}^{\prime }=\Lambda_{h}-\lambda_{h}S_{h}-u_{1}\left(t\right)S_{h}-\mu_{h}S_{h},\\ C^{\prime }=u_{1}\left(t\right)S_{h}-\left(1-\eta u_{2}\left(t\right)\right)\lambda_{h}C-u_{h}C,\\ I_{h}^{\prime }=\lambda_{h}S_{h}+\left(1-\eta u_{2}\left(t\right)\right)\lambda_{h}C-\left(\mu_{h}+\gamma \right)I_{h},\\ R_{h}^{\prime }=\gamma I_{h}-\mu_{h}R_{h},\\ S_{a}^{\prime }=\Lambda_{a}-\lambda_{a}S_{a}-\mu_{a}S_{a},\\ I_{a}^{\prime }=\lambda_{a}S_{a}-\left(\mu_{a}+\phi +v+\tau \right)I_{a},\\ R_{a}^{\prime }=\phi I_{a}-\mu_{a}R_{a},\\ W^{\prime }=\delta_{h}I_{h}+\delta_{a}I_{a}-rW. \end{array}\right. \end{array}$$

The term (1 − *ηu*_2_(*t*)) corresponds to the situation which prevents or limits the contacts between the susceptible humans and the infected environments and animals (they are reduced through necessary and efficient education about zoonotic diseases among the susceptible individuals), where *u*_2_(*t*) is the control. It is worth noting that smaller *η* implies less efficient educational campaign strategies in the communities (possibly due to, for example, individuals not being given enough and necessary information towards zoonotic diseases or not using the required language for that respective region). The ideal case, (1 − *ηu*_2_) ≈ 0, corresponds to a situation when the likelihood of being infected by the zoonotic disease is almost zero. Practically, this can be achieved by those who have managed to understand and are following the educational campaign information as per the health caregivers.

The goal is to minimize the number of infected humans over a finite time interval [0, *T*] at a minimal cost of effort, where *T* is the final time. Mathematically, we formulate our objective functional *ℱ* as follows:

(20) $$\begin{array}{c} F\left(u_{1},u_{2}\right)=\int_{0}^{T}\left[I_{h}+Au_{1}^{2}+Bu_{2}^{2}\right]\;dt. \end{array}$$

The control efforts are assumed to be nonlinear. We choose to model the control efforts using the quadratic terms, *u*_1_^2^(*t*), *u*_2_^2^(*t*), where the coefficients *A* and *B* represent weights in the cost of the controls. The weight constant over the prescribed time horizon is a measure of the relative costs of the intervention in connection with the reduction of infectious humans. Our problem is to find the optimal controls, *u*_1_^∗^(*t*) and *u*_2_^∗^(*t*), such that

(21) $$\begin{array}{c} F\left(u_{1}^{*},u_{2}^{*}\right)=\underset{\Omega }{\min}F\left(u_{1}\left(t\right),u_{2}\left(t\right)\right), \end{array}$$

where *Ω* = {*u*_1_, *u*_2_ ∈ *L*^1^(0, *T*)||0 ≤ *u*_1_, *u*_2_ ≤ 1, *t* ∈ [0, *T*]} subject to the state equations (19) with initial conditions. Given the criterion (20) and the regularity of the system of equation (19), the existence of optimal controls is guaranteed by standard results in control theory [41]. The necessary conditions that optimal solutions must satisfy are derived from Pontryagin's Maximum Principle [42]. This principle converts the system (19), (20), and (21) into the problem of minimizing the Hamiltonian *H* given by

(22) $$\begin{array}{c} H=I_{h}+Au_{1}^{2}+Bu_{2}^{2}+\lambda_{1}\left[\Lambda_{h}-\lambda_{h}S_{h}-u_{1}\left(t\right)S_{h}-\mu_{h}S_{h}\right]+\lambda_{2}\left[u_{1}\left(t\right)S_{h}-\left(1-\eta u_{2}\left(t\right)\right)\lambda_{h}C-\mu_{h}C\right]+\lambda_{3}\left[\lambda_{h}S_{h}+\left(1-\eta u_{2}\left(t\right)\right)\lambda_{h}C-\left(\mu_{h}+\gamma \right)I_{h}\right]+\lambda_{4}\left[\gamma I_{h}-\mu_{h}R_{h}\right]+\lambda_{5}\left[\Lambda_{a}-\lambda_{a}S_{a}-\mu_{a}S_{a}\right]+\lambda_{6}\left[\lambda_{a}S_{a}-\left(\mu_{a}+\phi +v+\tau \right)I_{a}\right]+\lambda_{7}\left[\phi I_{a}-\mu_{a}R_{a}\right]+\lambda_{8}\left[\delta_{h}I_{h}+\delta_{a}I_{a}-rW\right]. \end{array}$$

From this Hamiltonian and Pontryagin's Maximum Principle [42], we obtain the following theorem.

**Theorem 5 .**

There exist optimal controls *u*_1_^∗^, *u*_2_^∗^ and corresponding solutions, *S*_*h*_^*⋆*^, *C*^*⋆*^, *I*_*h*_^*⋆*^, *R*_*h*_^*⋆*^, *S*_*a*_^*⋆*^, *I*_*a*_^*⋆*^, *R*_*a*_^*⋆*^, and *W*^*⋆*^, that minimizes *ℱ*(*u*_1_(*t*), *u*_2_(*t*)) over *Ω*. In order for the above statement to be true, it is necessary that there exist continuous functions *λ*_*i*_(*t*), *i* = 1, 2, ⋯, 8 such that

(23) $$\begin{array}{c} \begin{array}{cccc} {\dot{\lambda }}_{1}\left(t\right)=\lambda_{h}\left[\lambda_{1}-\lambda_{3}\right]+u_{1}\left[\lambda_{1}-\lambda_{2}\right]+\mu_{h}\lambda_{1}, & & & \\ {\dot{\lambda }}_{2}\left(t\right)=\left(1-\eta u_{2}\right)\lambda_{h}\left[\lambda_{2}-\lambda_{3}\right]+\mu_{h}\lambda_{2}, & & & \\ {\dot{\lambda }}_{3}\left(t\right)=-1+\gamma \left[\lambda_{3}-\lambda_{4}\right]+\mu_{h}\lambda_{3}-\delta_{h}\lambda_{3}, & & & \\ {\dot{\lambda }}_{4}\left(t\right)=\mu_{h}\lambda_{4}, & & & \\ {\dot{\lambda }}_{5}\left(t\right)=\lambda_{4}\left[\lambda_{5}-\lambda_{6}\right]+\mu_{a}\lambda_{5}, & & & \\ {\dot{\lambda }}_{6}\left(t\right)=\frac{\beta_{h}S_{h}\left[\lambda_{1}-\lambda_{3}\right]}{N_{a}}+\frac{\left(1-\eta u_{2}\right)\beta_{h}C\left[\lambda_{2}-\lambda_{3}\right]}{N_{a}}+\frac{\beta_{a}S_{a}\left[\lambda_{5}-\lambda_{6}\right]}{N_{a}}+\phi \left[\lambda_{6}-\lambda_{7}\right]+\left(\mu_{a}+v+\tau \right)\lambda_{6}-\delta_{a}\lambda_{8}, & & & \\ {\dot{\lambda }}_{7}\left(t\right)=\mu_{a}\lambda_{7}, & & & \\ {\dot{\lambda }}_{8}\left(t\right)=\frac{\beta_{w_{h}}S_{h}\left[\lambda_{1}-\lambda_{3}\right]}{K}+\frac{\left(1-\eta u_{2}\right)\beta_{w_{h}}C\left[\lambda_{2}-\lambda_{3}\right]}{K}+\frac{\beta_{w_{a}}S_{a}\left[\lambda_{5}-\lambda_{6}\right]}{K}+r\lambda_{8}, & & & \end{array} \end{array}$$

with transversality conditions

(24) $$\begin{array}{c} \lambda_{i}=0,i=1,\cdots ,8. \end{array}$$

Furthermore, the optimal control is represented by

(25) $$\begin{array}{c} u_{1}^{*}\left(t\right)=\max\left\{0,\min\left(u_{\max},\frac{\left[\lambda_{1}-\lambda_{2}\right]S_{h}}{2A}\right)\right\},u_{2}^{*}\left(t\right)=\max\left\{0,\min\left(u_{\max},\frac{\left[\lambda_{3}-\lambda_{2}\right]{\eta \lambda }_{h}C}{2B}\right)\right\} \end{array}$$

**Proof**

The existence of optimal controls follows from Corollary 4.1 of [41] since the integrand of *ℱ* is a convex function of *u*_1_(*t*), *u*_2_(*t*) and the state system satisfies the *Lipshitz* property with respect to the state variables. The following can be derived from the Pontryagin's Maximum Principle [42]:

(26) $$\begin{array}{c} \frac{d\lambda_{1}}{dt}=-\frac{\partial H}{\partial S},\\ \frac{d\lambda_{2}}{dt}=-\frac{\partial H}{\partial C},\\ \frac{d\lambda_{3}}{dt}=-\frac{\partial H}{\partial I_{h}},\\ \frac{d\lambda_{4}}{dt}=-\frac{\partial H}{\partial R_{h}},\\ \frac{d\lambda_{5}}{dt}=-\frac{\partial H}{\partial S_{a}},\\ \frac{d\lambda_{6}}{dt}=-\frac{\partial H}{\partial I_{a}},\\ \frac{d\lambda_{7}}{dt}=-\frac{\partial H}{\partial R_{a}},\\ \frac{d\lambda_{8}}{dt}=-\frac{\partial H}{\partial W}, \end{array}$$

with *λ*_*i*_(*T*) = 0 for *i* = 1, ⋯, 8 evaluated at the optimal controls and corresponding states, which results in the adjoint system (23). The Hamiltonian *H* is minimized with respect to the controls at the optimal controls, so we differentiate *H* with respect to *u*_1_ and *u*_2_ on the set *Ω*, giving the following optimality conditions:

(27) $$\begin{array}{c} \frac{\partial H}{\partial u_{1}}=2Au_{1}+\left(\lambda_{2}-\lambda_{1}\right)S_{h}=0\text{ at }u_{1}=u_{1}^{*},\\ \frac{\partial H}{\partial u_{2}}=2Bu_{2}+\left(\lambda_{2}-\lambda_{3}\right)\eta C\lambda_{h}=0\text{ at }u_{2}=u_{2}^{*}. \end{array}$$

Solving for *u*_1_^∗^ and *u*_2_^∗^, we obtain

(28) $$\begin{array}{c} u_{1}^{*}=\frac{\left[\lambda_{1}-\lambda_{2}\right]S_{h}}{2A},\\ u_{2}^{*}=\frac{\left[\lambda_{3}-\lambda_{2}\right]\eta \lambda_{h}C}{2B}. \end{array}$$

By using the bounds 0 ≤ *u*_1_, *u*_2_ ≤ 1, we have the property (25).

Our optimal control problem thus couples the state system (19), the adjoint system (23), and the optimality condition (25). These equations are solved numerically using the forward-backward sweeping method, based on parameters and initial conditions listed in Table 1.

## 6. Numerical Simulations

In this section, we now present numerical simulations. The existence of optimal control is provided, and the behavior of the optimality system made of 8 ordinary differential equations is evaluated through numerical simulations done with Matlab. The optimality system is solved using an iterative method with Runge-Kutta fourth-order scheme. Starting with a guess for the adjoint variables, the state equations are solved forward in time. Then, these state values are used to solve the adjoint equations backward in time, and the iterations continue until convergence [39].

To illustrate the results of the foregoing analysis, we have simulated system (3) using the parameters in Table 1. We then assume some of the parameters in the realistic range for illustrative purposes. Among the estimated parameters, are the balancing coefficients that have been arbitrarily chosen for illustration purposes. These weight parameters determine the importance of variables in the objective functional (20), thus *A* = 1, *B* = 2.

**Figure 1:**
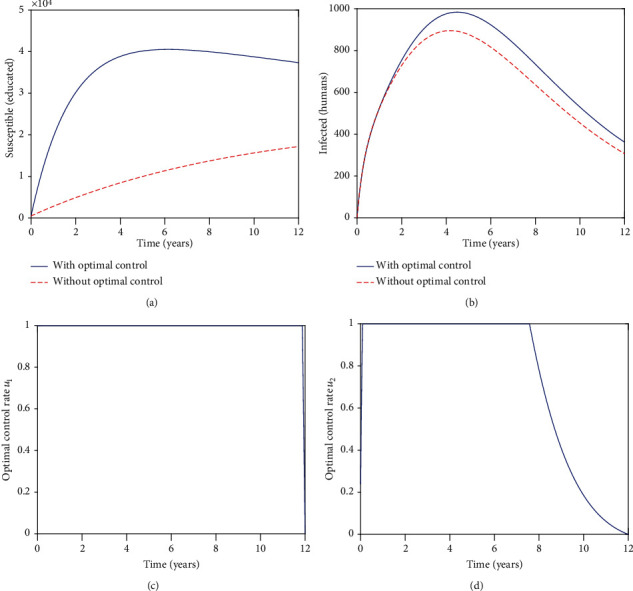
Graphs of the numerical solutions of the optimality system, showing the propagation of (a) the educated susceptible population *C* and (b) the infected human population *I*_*h*_; (c, d) showing the optimal control graphs for the two controls, *u*_1_ and *u*_2_, respectively. Over a period of 12 years with *η* = 0.25.

Figure 1(a) shows the impact of the controls on educating susceptible individuals towards zoonotic diseases. Both populations, in the presence and absence of controls, increase in the first 3 years. In the presence of controls, the population begins to drop gradually for the remainder of the period under study. After 3 years in the absence of controls, the population continues to increase steadily for the remainder of the period under review. We can see that in the presence of controls, we have more individuals being educated than in the absence of controls. It is worth noting that the controls are effective from the initial time.

Figure 1(b) depicts the impact of the controls on the infected human population. We note that when the educational campaign efficacy is low, the controls are not effective.

Figure 1(c) represents the control *u*_1_. The control *u*_1_ is initiated from the initial time. After initiation, the control is at the upper bound for the whole period under study.  Figure 1(d) represents the control *u*_2_. Control *u*_2_ is also initiated from the initial time. After initiation, the control *u*_2_ is at the upper bound for approximately 7 years, before returning to the lower bound, 0. Thus, control *u*_1_ is feasible for the whole period under review compared to control *u*_2_, which is feasible for only 7 years. In the presence of low educational campaign efficacy, control *u*_2_ is unsustainable.

**Figure 2:**
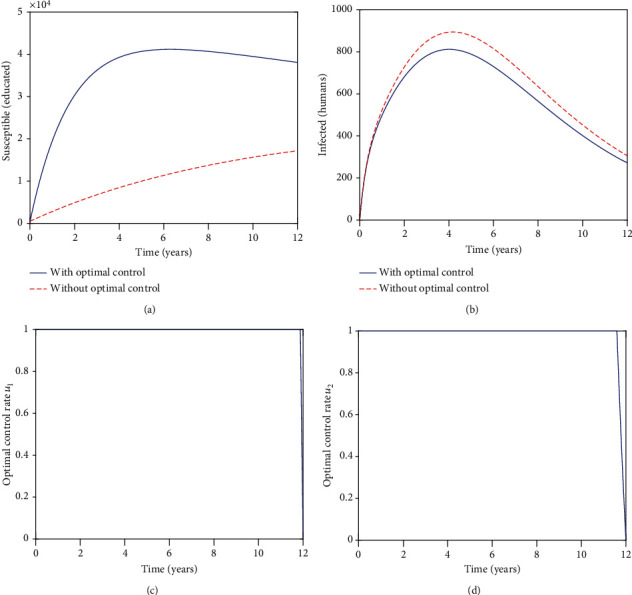
Graphs of the numerical solutions of the optimality system, showing the propagation of (a) the educated susceptible populations *C* and (b) the infected human population *I*_*h*_; (c, d) showing the optimal control graphs for the two controls, *u*_1_ and *u*_2_, respectively. Over a period of 12 years with *η* = 0.5.

Figure 2(a) illustrates the effects of the controls on educating susceptible individuals towards zoonotic diseases. Both populations, in the presence and absence of controls, increase in the first 3 years. In the presence of controls, the population begins to drop gradually for the remainder of the period under study. After 3 years in the absence of controls, the population continues to increase steadily for the remainder of the period under review. It is worth noting that in the presence of controls, we have more individuals being educated than in the absence of controls. Comparing Figures 1(a) and 2(a), we can see that an increase in *η* from 0.25 to 0.5, does not have much impact on educating more people towards zoonotic diseases.

Figure 2(b) illustrates the impact of the controls, on the infected human population. The controls start being effective from the initial time. We note that the controls slightly reduce the number of infected human individuals. Thus, increasing the efficacy of the educational campaigns has an impact on reducing the infected population. On comparing Figures 1(b) and 2(b), it is worth noting that increasing the efficacy of the educational campaigns results in the decrease of the infected humans.

Figure 2(c) represents the control *u*_1_, and Figure 2(d) represents the control *u*_2_. The control *u*_1_ is initiated from the initial time. After initiation, the control *u*_1_ is at the upper bound for the whole period under study. Control *u*_2_ is initiated from 0 years and stays at the upper bound for approximately 11 years. Thus, control *u*_1_ is feasible for the whole period under review compared to control *u*_2_ which is feasible for 11 years. Both controls are crucial.

**Figure 3:**
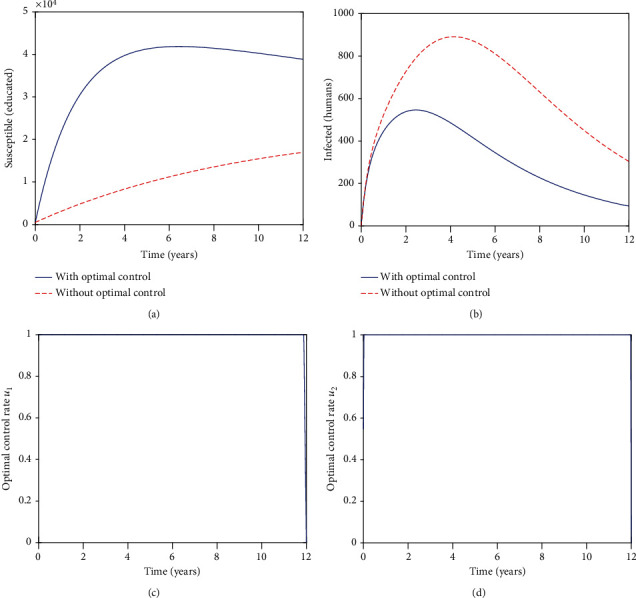
Graphs of the numerical solutions of the optimality system, showing the propagation of (a) the educated susceptible populations *C* and (b) the infected human population *I*_*h*_; (c, d) showing the optimal control graphs for the two controls, *u*_1_ and *u*_2_, respectively. Over a period of 12 years with *η* = 0.75.

Figure 3(a) highlights the impact of controls on the population of the educated susceptibles. The controls start being effective from the initial time. The population for the educated susceptibles increases for both cases (with and without controls). It is worth noting that, in the presence of controls, we have more educated individuals as compared to the absence of controls. Comparing Figures 1(a), 2(a), and 3(a), we note that for the scenario when the efficacy of educational campaigns is at 0.75, which is our maximum value, there is no much difference in the number of susceptible individuals who become educated on zoonotic diseases.

Figure 3(b) highlights the impact of controls on the population of infected individuals. Controls start being effective from the initial time. After 2 years, we can see that we have less infected individuals in the presence of controls as compared to the absence of controls. Thus, for *η* = 0.75, the cumulative cases for the infected individuals are less, compared to the populations when the values for the efficacy are *η* = 0.5 and *η* = 0.25.

Figure 3(c) represents the control *u*_1_, while Figure 3(d) illustrates the control *u*_2_. The results suggest that more effort should be devoted to both controls since they are both feasible for the whole period under study.

Overall, we can see that if the educational campaign's efficacy is high, *η* = 0.75, we have more individuals being educated and the maximum reduction of the individuals being infected by brucellosis. Furthermore, we note that the controls are both feasible and can be well implemented when the educational campaign's efficacy is high. It is also worth noting that, the efficacy of the educational campaigns can be increased without incurring any extra costs. Thus, health promotion programs need to be implemented with high efficacy for them to be effective. Comparing the weights in the cost of the controls, we have *B* > *A*; this implies that the costs incurred for making educational campaigns to have high efficacy are more than just conducting educational campaigns.

## 7. Discussion

Carrying out educational campaigns on zoonotic diseases within communities could help reduce the public health implications of zoonotic infections in human-domestic animal-wildlife interface areas, although it will come with certain costs. We have formulated and analyzed a differential equation-based deterministic model for zoonotic disease in the form of brucellosis, with the aim of assessing the impact of educational campaigns on curtailing the spread at minimal costs. The reproduction number is computed and shown that it is an important threshold quantity for disease dynamics. Through the construction of suitable Lyapunov functionals, it has been shown that the model has a stable disease-free equilibrium whenever the reproduction number is less than unity. It was shown that if the reproduction number is greater than unity, the disease persists. Further, it was also demonstrated that whenever the reproduction number is greater than unity, then the model has a unique endemic equilibrium point which is globally asymptotically stable. Sensitivity analysis of the model parameters was carried out, and it was noted that educating susceptible individuals towards zoonotic diseases was vital in the reduction of the infection. Also, the educational campaigns employed should have high efficacy. The model is further extended to incorporate two optimal intervention strategies. The major aim of the controls was to reduce the number of infected individuals at minimal costs. The goal of the first optimal intervention strategy aims to increase the rate of educating the susceptibles towards zoonotic diseases. The second control aims at increasing the positive impact of educating the susceptible individuals, that is, increases the efficacy of the educational campaigns. From the illustrations in our study, it is clear that the time-dependent intervention strategies can lead to the reduction of the zoonotic disease in the community. The efficacy of the educational campaigns is varied in the range of 0.25-0.75. Our findings illustrate that the larger the efficacy, the stronger the impact of both control strategies. Furthermore, we noted that for low and high efficacy levels, we obtained different results with the same costs. Thus, educational campaigns should be carried out with high efficacy. It is worth noting that there was no change in the costs, for implementing the controls when *η* = 0.25, *η* = 0.5, and *η* = 075. Comparing our results with others in the literature, it is worth noting that most of them focused on the impact of culling in the reduction of brucellosis prevalence. Some noted the importance of the elimination of the disease in domestic ruminants as the best control strategy. The aspect of vaccines was also investigated, and it was noted that vaccinating both the young and the old was vital. Thus, our results also provide an important avenue in the fight against brucellosis.

Our study has a few limitations. We had to base our numerical results from data which has been published in various literatures. More data sets and experimental studies are needed to include more realistic biological processes in the models. We assumed recruitment through birth only for both cattle and humans, and we left out migration; hence, no one joins the model as a susceptible educated individual. Constant populations have been assumed for both humans *N*_*h*_ and cattle *N*_*a*_. However, just like any other model, we cannot say the model is complete; it can be extended to include the aspect of the seasonality in the spread of the zoonotic disease.

## Figures and Tables

**Table 1:** Model variables.

Parameter	Baseline values
*S*_*h*_	Uneducated susceptible humans
*C*	Educated susceptible humans
*I*_*h*_	Infected humans
*R*_*h*_	Recovered humans
*S*_*a*_	Susceptible cattle
*I*_*a*_	Infected cattle
*R*_*a*_	Recovered cattle

**Table 2:** Model parameters and their baseline values. The time unit is a year.

Parameter	Definition	Baseline values	Source
*Λ*_*h*_	Recruitment rate (humans)	0.03	
*Λ*_*a*_	Recruitment rate (cattle)	0.83	
*μ*_*h*_	Natural death rate (humans)	0.02	[22]
*μ*_*a*_	Natural death rate (cattle)	0.04	[18]
*v*	Disease related death rate (cattle)	0.05	[18]
*τ*	Culling rate	0.15	[23]
*β*_*a*_	Cattle-to-cattle transmission	1.19	[18]
*β*_*wa*_	Brucella-to-cattle transmission	0.6	Assumed
*β*_*h*_	Cattle-to-human transmission	0.1	Assumed
*β*_*wh*_	Brucella-to-human transmission	0.05	Assumed
*δ*_*a*_	Brucella shedding by infected cattle	0.208	Assumed
*δ*_*h*_	Brucella shedding by infected humans	0.02	Assumed
*r*	Decay rate of brucella in the environment	3.6	[23]
*γ*	Recovery rate, cattle	0.208	[24]
*ϕ*	Recovery rate, humans	0.615	[25]
*η*	Efficacy of the health promotion programs	(0,1)	Assumed
*θ*	Rate of education	0.5	[7]

**Table 3:** Sensitivity indices of model parameters to *ℛ*_0_.

Parameter	Definition	Sensitivity index
*μ*_*h*_	Natural death rate (humans)	-0.107749
*μ*_*a*_	Natural death rate (cattle)	-0.657185
*v*	Disease related death rate (cattle)	-0.083362
*τ*	Culling rate	-0.504128
*β*_*a*_	Cattle-to-cattle transmission	0.377568
*β*_*wa*_	Brucella-to-cattle transmission	0.765102
*β*_*h*_	Cattle-to-human transmission	0.110828
*β*_*wh*_	Brucella-to-human transmission	0.046502
*δ*_*a*_	Brucella shedding by infected cattle	0.754275
*δ*_*h*_	Brucella shedding by infected humans	0.05733
*r*	Decay rate of brucella in the environment	-0.811605
*ϕ*	Recovery rate (cattle)	-0.047289
*γ*	Recovery rate (humans)	-0.105369
*η*	Efficacy of educational campaigns	-0.401162
*θ*	Rate of education	-0.792678

## Data Availability

The data used to support the findings of this study are included within the article and cited accordingly.

## Appendix

### A. Proof of Theorem 5

Proof To apply Lemma 4, the following simplifications and changes of variables are made first. Let *S*_*h*_ = *x*_1_, *C* = *x*_2_, *I*_*h*_ = *x*_3_, *R*_*h*_ = *x*_4_, *S*_*a*_ = *x*_5_, *I*_*a*_ = *x*_6_, *R*_*a*_ = *x*_7_, *W* = *x*_8_, so that *N*_*h*_ = *x*_1_ + *x*_2_ + *x*_3_ + *x*_4_ and *N*_*a*_ = *x*_5_ + *x*_6_ + *x*_7_. Further, by using the vector notation *X* = (*f*_1_, *f*_2_, *f*_3_, *f*_4_, *f*_5_, *f*_6_, *f*_7_, *f*_8_), then system (4) can be written in the form *dX*/*dt* = *F*(*x*), with *F* = (*f*_1_, *f*_2_, *f*_3_, *f*_4_, *f*_5_, *f*_6_, *f*_7_, *f*_8_)^*T*^, such that

(A.1) $$\begin{array}{c} \begin{array}{cccc} \frac{dx_{1}}{dt}={f_{1}}^{\prime }=\Lambda_{\text{h}}-\frac{\beta_{h}x_{1}x_{6}}{x_{5}+x_{6}+x_{7}}-\frac{\beta_{w_{h}}x_{1}x_{8}}{K}-\theta x_{1}-\mu_{h}x_{1}, & & & \\ \frac{d{\text{x}}_{2}}{dt}={f_{2}}^{\prime }=\theta x_{1}-\frac{\left(1-\eta \right)\beta_{h}x_{2}x_{6}}{x_{5}+x_{6}+x_{7}}-\frac{\left(1-\eta \right)\beta_{w_{h}}x_{2}x_{8}}{K}-\mu_{h}x_{2}, & & & \\ \frac{dx_{3}}{dt}={f_{3}}^{\prime }=\frac{\beta_{h}x_{1}x_{6}}{x_{5}+x_{6}+x_{7}}+\frac{\beta_{w_{h}}x_{1}x_{8}}{K}+\frac{\left(1-\eta \right)\beta_{h}x_{2}x_{6}}{x_{5}+x_{6}+x_{7}}+\frac{\left(1-\eta \right)\beta_{w_{h}}x_{2}x_{8}}{K}-\left(\gamma +\mu_{h}\right)x_{3},\\ \frac{dx_{4}}{dt}={f_{4}}^{\prime }=\gamma x_{3}-\mu_{h}x_{4},\\ \frac{dx_{5}}{dt}={f_{5}}^{\prime }=\Lambda_{a}-\frac{\beta_{a}x_{5}x_{6}}{x_{5}+x_{6}+x_{7}}-\frac{\beta_{w_{a}}x_{5}x_{8}}{K}-\mu_{a}x_{5}, & & & \\ \frac{dx_{6}}{dt}={f_{6}}^{\prime }=\frac{\beta_{a}x_{5}x_{6}}{x_{5}+x_{6}+x_{7}}+\frac{\beta_{w_{a}}x_{5}x_{8}}{K}-\left(\mu_{a}+\phi +v+\tau \right)x_{6}, & & & \\ \frac{dx_{7}}{dt}={f_{7}}^{\prime }=\phi x_{6}-\mu_{a}x_{7},\\ \frac{dx_{8}}{dt}={f_{8}}^{\prime }=\delta_{h}x_{3}+\delta_{a}x_{6}-rx_{8}. & & & \end{array} \end{array}$$

The Jacobian matrix of system (A.1) at *ℰ*^0^ is given by

(A.2) $$\begin{array}{c} \left(\begin{array}{cccccccc} -\left(\theta +\mu_{h}\right) & 0 & 0 & 0 & 0 & -\frac{\beta_{h}\mu_{a}\Lambda_{h}}{\Lambda_{a}\left(\theta +\mu_{h}\right)} & 0 & -\frac{\beta_{w_{h}}\Lambda_{h}}{\left(\theta +\mu_{h}\right)K}\\ \theta & -\mu_{h} & 0 & 0 & 0 & -\frac{\beta_{h}\left(1-\eta \right)\theta \Lambda_{h}\mu_{a}}{\mu_{h}\left(\theta +\mu_{h}\right)\Lambda_{a}} & 0 & -\frac{\beta_{w_{h}}\left(1-\eta \right)\theta \Lambda_{h}}{\mu_{h}\left(\theta +\mu_{h}\right)K}\\ 0 & 0 & \gamma & 0 & 0 & \frac{\beta_{h}\mu_{a}\Lambda_{h}}{\Lambda_{a}\left(\theta +\mu_{h}\right)}+\frac{\beta_{h}\left(1-\eta \right)\theta \Lambda_{h}\mu_{a}}{\mu_{h}\left(\theta +\mu_{h}\right)\Lambda_{a}} & 0 & \frac{\beta_{w_{h}}\Lambda_{h}}{\left(\theta +\mu_{h}\right)K}+\frac{\beta_{w_{h}}\left(1-\eta \right)\theta \Lambda_{h}}{\mu_{h}\left(\theta +\mu_{h}\right)K}\\ 0 & 0 & \gamma & -\mu_{h} & 0 & 0 & 0 & 0\\ 0 & 0 & 0 & 0 & -\mu_{a} & -\beta_{a} & 0 & \frac{\beta_{w_{a}}\mu_{a}}{\Lambda_{a}K}\\ 0 & 0 & 0 & 0 & 0 & \beta_{a}-\left(\mu_{a}+\phi +v+\tau \right) & 0 & -\frac{\beta_{w}\mu_{a}}{\Lambda_{a}K}\\ 0 & 0 & 0 & 0 & 0 & \phi & -\mu_{a} & 0\\ 0 & 0 & \delta_{h} & 0 & 0 & \delta_{a} & 0 & -r \end{array}\right) \end{array}$$

from which it can be shown that

(A.3) $$\begin{array}{c} R_{0}=\rho \left(FV^{-1}\right)=\frac{A_{11}+A_{22}+\sqrt{{\left(A_{11}-A_{22}\right)}^{2}+4A_{12}A_{21}}}{2}, \end{array}$$

with *A*_11_, *A*_12_, *A*_21_, *A*_22_ as defined in equation (8). Now, we consider *ρ*_1_*β*_*h*_ = *β*_*w*_*h*__, *ρ*_2_*β*_*h*_ = *β*_*a*_, *ρ*_3_*β*_*h*_ = *β*_*w*_*a*__, regardless of whether *ρ*_1_, *ρ*_2_, *ρ*_3_ ∈ (0, 1) or *ρ*_1_, *ρ*_2_, *ρ*_3_ ≥ (0, 1). Taking *β*_*h*_ as the bifurcation parameter and considering that *ℛ*_0_ = 1 and solving for *β*_*h*_, we have

(A.4) $$\begin{array}{c} \beta^{*}=\beta_{h}=\frac{2}{{A_{11}}^{\prime }+{A_{22}}^{\prime }+\sqrt{{\left({A_{11}}^{\prime }-{A_{22}}^{\prime }\right)}^{2}+4{A_{12}}^{\prime }{A_{21}}^{\prime }}}, \end{array}$$

where

(A.5) $$\begin{array}{c} A_{11}^{\prime }=\frac{\left(\left(1-\eta \right)\theta +\mu_{h}\right)\delta_{h}\rho_{1}\Lambda_{h}}{\left(\gamma +\mu_{h}+v\right)\left(\theta +\mu_{h}\right)\mu_{h}rK},\\ {A_{12}}^{\prime }=\frac{\left(\left(1-\eta \right)\theta +\mu_{h}\right)\left(\rho_{1}\delta_{a}\Lambda_{a}+\mu_{a}rK\right)\Lambda_{h}}{\mu_{h}rK\left(\theta +\mu_{h}\right)\left(\phi +\mu_{a}+v+\tau \right)\Lambda_{a}},\\ A_{21}^{\prime }=\frac{\delta_{h}\rho_{3}\Lambda }{\left(\gamma +\mu_{h}\right)\mu_{a}rK},\\ {A_{22}}^{\prime }=\frac{\mu_{a}\rho_{2}rK+\delta_{a}\rho_{3}\Lambda_{a}}{\left(\phi +\mu_{a}+v+\tau \right)\mu_{a}rK}. \end{array}$$

Note that the linearised system of the transformed equation (A.1) with the bifurcation point *β*^∗^ has a zero eigenvalue. Hence, the Centre Manifold Theory [36] can be used to analyze the dynamics of system (A.1) near *β*_*h*_ = *β*^∗^. It can be shown that the Jacobian of system (A.1) has a right eigenvector associated with the following zero eigenvalue given by *u* = (*u*_1_, *u*_2_, *u*_3_, *u*_4_, *u*_5_, *u*_6_, *u*_7_, *u*_8_)^*T*^, where

(A.6) $$\begin{array}{c} u_{1}=-\frac{\mu_{a}}{\theta +\mu_{h}}\left[\frac{\beta_{w_{h}}\Lambda_{h}\delta_{h}\mu_{h}}{\mu_{a}\left(\theta +\mu_{h}\right)r\gamma K}u_{4}+\left(\frac{\beta_{h}\Lambda \mu_{a}}{\phi \left(\theta +\mu_{h}\right)\Lambda_{a}}+\frac{\theta \Lambda_{h}\beta_{w_{h}}\delta_{a}}{\mu_{a}\left(\theta +\mu_{h}\right)r\phi K}\right)u_{7}\right]<0,\\ u_{2}=-\frac{1}{\mu_{h}}\left[\frac{\left(1-\eta \right)\beta_{w_{h}}\Lambda_{h}\theta \delta_{h}\mu_{h}}{\mu_{h}\left(\theta +\mu_{h}\right)r\gamma K}u_{4}+\left(\frac{\left(1-\eta \right)\beta_{h}\theta \Lambda_{h}\mu_{a}}{\phi \left(\theta +\mu_{h}\right)\Lambda_{a}\mu_{h}}+\frac{\left(1-\eta \right)\theta \Lambda_{h}\beta_{w_{h}}\delta_{a}\mu_{a}}{\mu_{h}\left(\theta +\mu_{a}\right)r\phi K}\right)u_{7}\right]<0,\\ u_{5}=-\frac{1}{\mu_{a}}\left[\frac{\beta_{w_{a}}\Lambda_{a}\delta_{h}\mu_{h}}{\mu_{a}rK}u_{4}+\left(\frac{\beta_{a}\mu_{a}}{\phi }+\frac{\Lambda_{a}\beta_{w_{a}}\delta_{a}}{r\phi K}\right)u_{7}\right]<0,\\ u_{3}=\frac{\mu_{h}}{\gamma }u_{4}>0,\\ u_{4}=u_{4}>0,\\ u_{6}=\frac{\mu_{a}}{\phi }u_{7}>0,\\ u_{7}=u_{7}>0,\\ u_{8}=\frac{\delta_{h}\mu_{h}}{r\gamma }u_{4}+\frac{\delta_{a}\mu_{a}}{r\phi }u_{7}>0. \end{array}$$

The left eigenvectors of *J*(*ℰ*^0^) associated with the zero eigenvalue at *β*_*h*_ = *β*^∗^ is given by

(A.7) $$\begin{array}{c} w={\left(w_{1},w_{2},w_{3},w_{4},w_{5},w_{6},w_{7},w_{8}\right)}^{T}, \end{array}$$

where

(A.8) $$\begin{array}{c} w_{1}=w_{2}=w_{4}=w_{5}=w_{7}=0,\\ w_{3}=\frac{\delta_{h}}{\gamma +\mu_{h}}w_{8},w_{8}=w_{8}>0,\\ w_{6}=\left(\frac{\mu_{a}\delta_{h}\left(\mu_{h}\beta_{h}\Lambda_{h}+\left(1-\eta \right)\beta_{h}\theta \Lambda_{h}\right)}{\mu_{h}\Lambda_{a}\left(\theta +\mu_{h}\right)\left(\gamma +\mu_{h}\right)\left(\left(\mu_{a}+\phi +v+\tau \right)-\beta_{a}\right)}\right)w_{8}. \end{array}$$

### B. Computation of the Bifurcation Parameters *a* and *b*

For the sign of *a*, it can be shown that the associated nonvanishing partial derivatives of *F* are given by

(A.9) $$\begin{array}{c} \begin{array}{cccc} \frac{\partial^{2}f_{3}}{\partial x_{1}\partial x_{6}}=\frac{\partial^{2}f_{3}}{\partial x_{6}\partial x_{1}}=\frac{\beta_{h}\mu_{a}}{\Lambda_{a}},\frac{\partial^{2}f_{3}}{\partial x_{1}\partial x_{8}}=\frac{\partial^{2}f_{3}}{\partial x_{8}\partial x_{1}}=\frac{\beta_{w_{h}}}{K},\frac{\partial^{2}f_{3}}{\partial x_{2}\partial x_{6}}=\frac{\partial^{2}f_{3}}{\partial x_{6}\partial x_{2}}=\frac{\left(1-\eta \right)\beta_{h}\mu_{a}}{\Lambda_{a}}, & & & \\ \frac{\partial^{2}f_{3}}{\partial x_{2}\partial x_{8}}=\frac{\partial^{2}f_{3}}{\partial x_{8}\partial x_{2}}=\frac{\left(1-\eta \right)\beta_{w_{h}}}{K},\frac{\partial^{2}f_{3}}{\partial x_{5}\partial x_{6}}=\frac{\partial^{2}f_{3}}{\partial x_{6}\partial x_{5}}=\frac{-\beta_{h}\Lambda_{h}\mu_{a}^{2}}{\left(\theta +\mu_{h}\right)\Lambda_{a}^{2}}\left[\frac{\theta \left(1-\eta \right)}{\mu_{h}}+1\right],\\ \frac{\partial^{2}f_{3}}{\partial x_{6}^{2}}=\frac{-2\beta_{h}\Lambda_{h}\mu_{a}^{2}}{\Lambda_{a}\left(\theta +\mu_{h}\right)}\left[\frac{\theta \left(1-\eta \right)}{\mu_{h}}+1\right],\frac{\partial^{2}f_{3}}{\partial x_{6}\partial x_{7}}=\frac{\partial^{2}f_{3}}{\partial x_{7}\partial x_{6}}=\frac{\beta_{h}\Lambda_{h}}{\left(\theta +\mu_{h}\right)}+\frac{\beta_{h}\theta \Lambda_{h}\left(1-\eta \right)\mu_{a}}{\Lambda_{a}},\\ \frac{\partial^{2}f_{6}}{\partial x_{5}\partial x_{6}}=\frac{\partial^{2}f_{6}}{\partial x_{6}\partial x_{5}}=\frac{\beta_{wa}\mu_{a}}{\Lambda_{a}}\left[1-\frac{\mu_{a}}{\Lambda_{a}}\right],\frac{\partial^{2}f_{6}}{\partial x_{5}\partial x_{8}}=\frac{\partial^{2}f_{6}}{\partial x_{8}\partial x_{5}}=\frac{\beta_{wa}\mu_{a}}{K},\frac{\partial^{2}f_{6}}{\partial x_{5}\partial x_{6}^{2}}=\frac{-\beta_{a}\mu_{a}}{\Lambda_{a}},\\ \frac{\partial^{2}f_{6}}{\partial x_{6}\partial x_{7}}=\frac{\partial^{2}f_{6}}{\partial x_{7}\partial x_{6}}=\frac{-\beta_{wa}\mu_{a}}{\Lambda_{a}},\frac{\partial^{2}f_{3}}{\partial x_{6}\partial x_{7}}=\frac{\partial^{2}f_{3}}{\partial x_{7}\partial x_{6}}=\frac{-\beta_{h}\Lambda_{h}\mu_{a}^{2}}{\left(\theta +\mu_{h}\right)\Lambda_{a}^{2}}\left[\frac{\theta \left(1-\eta \right)}{\mu_{h}}+1\right]. \end{array} \end{array}$$

From (A.9), it follows that

(A.10) $$\begin{array}{c} \begin{array}{cc} a=\left[\beta_{h}\left(u_{1}+\left(1-\eta \right)u_{2}\right)\left(\frac{\mu_{a}u_{6}}{\Lambda }+\frac{\rho_{1}u_{8}}{K}\right)-\left(\frac{2\beta_{h}\mu_{a}u_{6}}{\Lambda_{a}}\left(u_{6}+u_{7}\right)+\frac{2\beta_{h}u_{5}u_{6}\mu_{a}^{2}}{\Lambda_{a}^{2}}\right)\left(\frac{\Lambda_{h}\left(\mu_{h}+\left(1-\eta \right)\theta \right)}{\mu_{h}\left(\mu_{h}+\theta \right)}\right)\right]w_{3}+\left[\frac{\beta_{h}u_{5}u_{8}\rho_{3}}{K}-\frac{\beta_{h}\mu_{a}u_{6}\rho_{2}}{\Lambda_{a}}\left(u_{6}+u_{7}\right)\right]w_{6}<0. & \end{array} \end{array}$$

This excludes the possibility of a backward bifurcation since *a* < 0. For the sign of *b*, it is associated with the following nonvanishing partial derivatives of *F*:

(A.11) $$\begin{array}{c} \frac{\partial^{2}f_{3}}{\partial x_{6}\partial \beta^{*}}=\frac{\mu_{a}\Lambda_{h}\left(\mu_{h}+\left(1-\eta \right)\theta \right)}{\mu_{h}\left(\mu_{h}+\theta \right)\Lambda_{a}},\\ \frac{\partial^{2}f_{3}}{\partial x_{8}\partial \beta^{*}}=\frac{\rho_{1}\Lambda_{h}\left(\mu_{h}+\left(1-\eta \right)\theta \right)}{\mu_{h}\left(\mu_{h}+\theta \right)K},\\ \frac{\partial^{2}f_{6}}{\partial x_{6}\partial \beta^{*}}=\rho_{2},\frac{\partial^{2}f_{6}}{\partial x_{8}\partial \beta^{*}}=\frac{\rho_{3}\mu_{a}}{K\Lambda_{a}}. \end{array}$$

It follows from the expressions in (A.11) that

(A.12) $$\begin{array}{c} b=\frac{\Lambda_{h}\left(\mu_{h}+\left(1-\eta \right)\theta \right)}{\mu_{h}\left(\mu_{h}+\theta \right)}\left(\frac{\mu_{a}u_{6}}{\Lambda_{a}}+\frac{\rho_{1}u_{8}}{K}\right)w_{3}+\frac{\Lambda_{a}}{\mu_{a}}\left(\frac{\rho_{2}\mu_{a}u_{6}}{\Lambda_{a}}+\frac{\rho_{3}u_{8}}{K}\right)w_{6}>0. \end{array}$$

Thus, *a* < 0 and *b* > 0 and applying Lemma 4 item (iv), we have established the following result.

Theorem 6 . The unique endemic equilibrium *ℰ*^∗^ is locally asymptotically stable for *ℛ*_0_ > 1, but close to 1.

## References

[1] Belay E. D., Kile J. C., Hall A. J. (2017). Zoonotic disease programs for enhancing global health security. *Emerging Infectious Diseases*.

[2] Grace D., Mutua F., Ochungo P. (2012). *Mapping of poverty and likely zoonoses hotspots*.

[3] Centers for Disease Control and Prevention (2017). National Center for Emerging and Zoonotic Infectious Diseases (NCEZID). *One Health*.

[4] Allen L. J. S., Allen V. L., Jonsson C. B. (2012). Mathematical MODELING of viral zoonoses in wildlife. *Natural Resource Modeling*.

[5] Morse S. S., Mazet J. A. K., Woolhouse M. (2012). Prediction and prevention of the next pandemic zoonosis. *The Lancet*.

[6] Kemuto N., Mogoa E., Osoro E., Bitek A., Njenga M. K., Thumbi S. M. (2018). Zoonotic disease research in East Africa. *BMC Infectious Disease*.

[7] Gadaga B. M., Etter E. M. C., Mukamuri B., Makwangudze K. J., Pfukenyi D. M., Matope G. (2015). Living at the edge of an interface area in Zimbabwe: cattle owners, commodity chain and health workers’ awareness, perceptions and practices on zoonoses. *BMC Public Health*.

[8] Sandip R., Terry F. M., Yan W. (2011). A network control theory approach to modeling and optimal control of zoonoses: case study of brucellosis transmission in sub-Saharan Africa. *PLoS Neglected Tropical Diseases*.

[9] Alexander K. A., Lewis B. L., Marathe M., Eubank S., Blackburn J. K. (2012). Modeling of wildlife-associated zoonoses: applications and caveats. *Vector-Borne and Zoonotic Diseases*.

[10] Royce K. P., Fu F. (2019). Mathematically modeling spillover dynamics of emerging zoonoses with intermediate hosts. https://arxiv.org/abs/1908.10791.

[11] Grant C., Iacono G. L., Dzingirai V., Bett B., Winnebah T. R. A., Atkinson P. M. (2016). Moving interdisciplinary science forward: integrating participatory modelling with mathematical modelling of zoonotic disease in Africa. *Infectious Diseases of Poverty*.

[12] Waters E. K., Hamilton A. J., Sidhu H. S., Sidhu L. A., Dunbar M. (2016). Zoonotic transmission of waterborne disease: a mathematical model. *Bulletin of Mathematical Biology*.

[13] Ambrose M. R., Kucharski A. J., Formenty P., Muyembe-Tamfum J., Rimoin A. W., Lloyd-Smith J. O. (2019). Quantifying transmission of emerging zoonoses: using mathematical models to maximize the value of surveillance data. *BioRxiv*.

[14] Nannyonga B., Mwanga G. G., Luboobi L. S. (2015). An optimal control problem for ovine brucellosis with culling. *Journal of Biological Dynamics*.

[15] Nyerere N., Luboobi L. S., Mpeshe S. C., Shirima G. M. (2019). Mathematical model for the infectiology of brucellosis with some control strategies. *New Trends in Mathematical Sciences*.

[16] Nyerere N., Luboobi L. S., Mpeshe S. C., Shirima G. M. (2019). Mathematical model for brucellosis transmission dynamics in livestock and human populations. *Communications in Mathematical Biology and Neuroscience*.

[17] Godfroid J. (2002). Brucellosis in wildlife. *Revue scientifique et technique (International Office of Epizootics)*.

[18] Abatih E., Ron L., Speybroeck N., Williams B., Berkvens D. (2015). Mathematical analysis of the transmission dynamics of brucellosis among bison. *Mathematical Methods in the Applied Sciences*.

[19] Braurer F., Chavez C. C. (2001). *Mathematical Models in Population Biology and Epidemiology*.

[20] Murray J. D. (2000). *Mathematical Biology I: An Introduction*.

[21] Lolika P. O., Modnak C., Mushayabasa S. (2018). On the dynamics of brucellosis infection in bison population with vertical transmission and culling. *Mathematical Biosciences*.

[22] Bhunu C. P., Mhlanga A., Mushayabasa S. (2014). Exploring the impact of prostitution on HIV/AIDS transmission. *International Scholarly Research Notices*.

[23] Hou Q., Sun X., Zhang J., Liu Y., Wang Y., Jin Z. (2013). Modeling the transmission dynamics of sheep brucellosis in Inner Mongolia Autonomous Region, China. *China, Mathematical Biosciences*.

[24] Li M., Sun G., Wu Y., Zhang J., Jin Z. (2014). Transmission dynamics of a multi-group brucellosis model with mixed cross infection in public farm. *Applied Mathematics and Computation*.

[25] Shi Y., Gao H., Pappas G. (2018). Clinical features of 2041 human brucellosis cases in China. *PLoS One*.

[26] Van den Driessche P., Watmough J. (2002). Reproduction numbers and sub-threshold endemic equilibria for compartmental models of disease transmission. *Mathematical Biosciences*.

[27] Korobeinikov A. (2004). Lyapunov functions and global properties for SEIR and SEIS epidemic models. *Mathematical Medicine and Biology*.

[28] McCluskey C. C. (2006). Lyapunov functions for tuberculosis models with fast and slow progression. *Mathematical Biosciences and Engineering*.

[29] Mukandavire Z., Chiyaka C., Magombedze G., Musuka G., Malunguza N. J. (2009). Assessing the effects of homosexuals and bisexuals on the intrinsic dynamics of HIV/AIDS in heterosexual settings. *Mathematical and Computer Modeling*.

[30] Shuai Z., Heesterbeek J. A. P., van den Driessche P. (2013). Extending the type reproduction number to infectious disease control targeting contacts between types. *Journal of Mathematical Biology*.

[31] Mhlanga A. (2019). Dynamical analysis and control strategies in modelling Ebola virus disease. *Advances in Difference Equations*.

[32] Horn R. A., Johnson C. R. (1985). *Matrix Analysis*.

[33] LaSalle J. P. (1976). *The stability of dynamical systems, in: CBMS-NSF regional conference series in applied mathematics*.

[34] Zhao X. Q. (2013). *Dynamical Systems in Population Biology*.

[35] Castillo-Chavez C., Song B. (2004). Dynamical models of tuberculosis and their applications. *Mathematical Biosciences and Engineering*.

[36] Powell D. R., Fair J., Le Claire R. J., Moore L. M., Thompson D. Sensitivity analysis of an infectious disease model.

[37] Arriola L. M., Hyman J. M. (2007). Being sensitive to uncertainty. *Computing in Science and Engineering*.

[38] Modnak C., Wang J., Mukandavire Z. (2014). Simulating optimal vaccination times during cholera outbreaks. *International Journal of Biomathematics*.

[39] Lenhart S., Workman J. T. (2007). *Optimal Control Applied to Biological Models*.

[40] Mhlanga A., Bhunu C. P., Mushayabasa S. (2015). A computational study of HSV-2 with poor treatment adherence. *Abstract and Applied Analysis*.

[41] Fleming W. H., Rishel R. W. (1975). *Deterministic and Stochastic Optimal Control*.

[42] Pontryagin L. S., Boltyanskii V. T., Gamkrelidze R. V., Mishchevko E. F. (1962). *The Mathematical Theory of Optimal Processes*.

